# Authentication, access, and monitoring system for critical areas with the use of artificial intelligence integrated into perimeter security in a data center

**DOI:** 10.3389/fdata.2023.1200390

**Published:** 2023-08-31

**Authors:** William Villegas-Ch, Joselin García-Ortiz

**Affiliations:** Escuela de Ingeniería en Ciberseguridad, Facultad de Ingenierías y Ciencias aplicada, Universidad de Las Américas, Quito, Ecuador

**Keywords:** artificial intelligence, computer vision, data center, information security, perimeter security

## Abstract

Perimeter security in data centers helps protect systems and the data they store by preventing unauthorized access and protecting critical resources from potential threats. According to the report of the information security company SonicWall, in 2021, there was a 66% increase in the number of ransomware attacks. In addition, the message from the same company indicates that the total number of cyber threats detected in 2021 increased by 24% compared to 2019. Among these attacks, the infrastructure of data centers was compromised; for this reason, organizations include elements Physical such as security cameras, movement detection systems, authentication systems, etc., as an additional measure that contributes to perimeter security. This work proposes using artificial intelligence in the perimeter security of data centers. It allows the automation and optimization of security processes, which translates into greater efficiency and reliability in the operations that prevent intrusions through authentication, permit verification, and monitoring critical areas. It is crucial to ensure that AI-based perimeter security systems are designed to protect and respect user privacy. In addition, it is essential to regularly monitor the effectiveness and integrity of these systems to ensure that they function correctly and meet security standards.

## 1. Introduction

Currently, the security of computer infrastructures goes beyond applications and services, as network experts seek to improve physical security in their networks (Morales et al., 2020). Therefore, the protection of the perimeter of a computer system is considered one of the most relevant aspects in the field of information security. Perimeter security aims to protect the system perimeter against intruders and is regarded as an organization's first line of defense (Mi et al., 2021). The importance of perimeter security is that any breach or vulnerability in this area can lead to unauthorized intrusions, putting data integrity and business continuity at risk (Lyu et al., 2021b). In this sense, current perimeter security systems include different layers of protection, such as video surveillance systems, access control, and intrusion detection systems ( Костюк and Коробейникова, 2021). However, despite advances in perimeter security technologies, persistent challenges need to be addressed. Among them are the high costs associated with deploying and maintaining sophisticated systems, the need to adapt to technological advances and constantly evolving threats, and the complexity of managing and monitoring multiple security systems.

In this context, this article focuses on addressing these challenges and providing an efficient solution to improve the perimeter security of computer systems (Dejdar et al., 2020). Our proposal focuses on developing algorithms based on artificial intelligence techniques that allow more efficient and effective management of perimeter security systems (Morales et al., 2020; Lai et al., 2022). The main objective of this article is to present an innovative approach to improve perimeter security, providing a solution that is effective, accessible, and scalable (Zhu et al., 2020). Our proposal uses facial recognition systems as an authentication method, taking advantage of this technology in terms of convenience, precision, and resistance to possible identity theft attempts.

In addition, our proposal focuses on taking advantage of the precision and security of facial recognition to improve the perimeter security of computer systems. Facial recognition systems offer additional protection against possible attempts to impersonate or falsify identity, providing excellent reliability in the authentication process (Bryson and Andres, 2020). In this work, the use of advanced facial recognition techniques is considered to guarantee the authenticity of users and strengthen perimeter security. Developing these algorithms based on artificial intelligence represents a significant advance in the field of perimeter security since it allows optimizing the use of resources and reducing associated costs. Also, being a scalable solution, it can be adapted to different environments and organizations, from small businesses to large corporations.

The results obtained in this work demonstrate the effectiveness and benefits of our proposal in terms of perimeter security. By implementing algorithms based on artificial intelligence techniques, we can improve the detection and prevention of possible threats in the perimeter of a data center. Facial recognition systems and verification of roles and privileges have proven to be highly effective in guaranteeing secure access to the different areas of the data center. Furthermore, our proposal has managed to reduce the costs associated with implementing traditional solutions by using the existing infrastructure and devices in the network architecture. These results demonstrate the potential of emerging technologies in conventional environments, enabling the creation of self-contained environments that provide secure access and strong protection in all areas of the data center.

## 2. Materials and methods

For the development of the method, several concepts are considered that are the fundamental basis for using techniques such as AI and computer vision. In addition, an analysis of existing similar works in perimeter security in critical areas has been carried out. This proposal uses a methodology based on a combination of techniques and approaches to carry out the relinking process in the context of perimeter security. In the first stage, the existing infrastructure is analyzed and evaluated; here, exhaustive research on the existing perimeter security infrastructure is carried out in the critical area under study. This implies reviewing device configurations, implemented security policies, intrusion detection, and response mechanisms, among other relevant aspects.

Next, identify vulnerabilities and weak points using security evaluation techniques, such as penetration tests and risk analysis; we identify possible vulnerabilities and weak points in the perimeter security infrastructure. This allows us to understand the areas that require improvement and reinforcement. In the next stage, the design and planning of the relinking are carried out; based on the findings of the infrastructure analysis and the identification of vulnerabilities, we proceed to design a relinking strategy that addresses the deficiencies detected. This involves selecting the right solutions and technologies to strengthen perimeter security.

Once the relinking strategy has been designed, we implement the proposed solutions. This may involve configuring new security devices, updating security policies, and integrating advanced detection and response systems, among other actions. Next, a testing and validation stage is entered, where exhaustive tests are carried out to evaluate the effectiveness of the implemented relinking solutions. This involves simulating different attack scenarios, monitoring security metrics, and verifying the integrity and confidentiality of perimeter communications.

Finally, all relevant aspects of the relinking process are documented, including a detailed description of the methodology used. This allows us to have a complete record of the actions carried out and facilitates the continuous monitoring and maintenance of perimeter security.

### 2.1. Proposed solution

The proposed solution uses computer vision systems and machine learning algorithms, effectively combined to improve perimeter security in data centers. In the development, a computer vision system is implemented to monitor and analyze the perimeter areas of the data center. These systems use cameras and sensors to capture images and data in real-time. We can detect patterns and abnormal behavior that could indicate a threat using advanced image processing techniques. In addition, we apply machine learning algorithms, specifically in facial recognition and verification of roles and privileges. These algorithms accurately identify individuals attempting to access the data center and verify their authorization levels. We use large and diverse data sets to train the algorithms, ensuring high accuracy in identity authentication.

Our solution integrates with existing security systems in the data center, such as access control systems and alarm systems. This allows for an immediate response to potential security breaches. When a threat or suspicious activity is detected, our system generates alerts in real-time and notifies those responsible for security, facilitating quick decision-making and action. The implement additional security measures to ensure our systems' integrity and protection. This includes image data encryption, user authentication and authorization to access information, and implementing attack detection techniques.

Taken together, the proposed solution significantly improves data center perimeter security. By using computer vision systems and machine learning algorithms, we achieve more accurate detection and faster response to potential threats. Integration with existing systems and real-time alert generation provides an additional layer of protection. In addition, our security considerations guarantee the integrity and confidentiality of the data.

### 2.2. Review of similar works

Several research papers make significant contributions to the field of perimeter security in critical areas. Security is strengthened by addressing issues such as cryptographic algorithm optimization, efficient implementation in embedded systems, and fault detection, and sensitive communications are protected in perimeter environments. This review has considered studies with a similar approach to our proposal.

This consideration mentions (Jalali et al., 2019a), work focuses on improving the security and efficiency of the Diffie-Hellman key exchange using supersingular isogenies on 64-bit ARM devices. Their contribution is especially relevant in environments with limited resources, such as embedded systems, where perimeter security is crucial. Furthermore, in Anastasova et al. (2021), the authors propose fast strategies for the efficient implementation of the SIKE Round 3 cryptographic scheme on ARM Cortex-M4 devices. By optimizing the performance of this scheme in systems with resource constraints, perimeter security is strengthened in critical areas, guaranteeing adequate protection of communications.

In Jalali et al. (2019b), he focuses on optimization and constant time in implementing the CSIDH scheme on embedded devices. Addressing performance and security challenges helps ensure the integrity of communications in critical areas and protects them from potential cyberattacks. Similarly, in Bisheh-Niasar et al. (2021) the development of cryptographic accelerators for digital signatures based on the Ed25519 scheme is proposed. These accelerators improve the efficiency and security of cryptographic operations in real-time, essential to guarantee perimeter security in critical areas and protect sensitive communications.

Another work such as Mozaffari-Kermani and Reyhani-Masoleh (2009), deals with the design of fault detection structures for S-boxes and the reverse S-boxes of the Advanced Encryption Standard (AES). Detecting and mitigating potential AES failures, you help strengthen perimeter security in critical areas and protect sensitive communications. For his part, in Dubrova et al. (2022), he focuses on the analysis of a copy-and-paste attack on a masked implementation of CRYSTALS -Kyber. Identifying and demonstrating a vulnerability in this cryptographic scheme highlights the importance of strengthening performances in critical areas to ensure perimeter security.

Finally, in Kermani and Azarderakhsh (2018), reliable error detection schemes for secure Galois Countermeasures Mode (GCM) cryptographic structures without dependency on architecture. By providing robust error detection mechanisms, perimeter security, and communications integrity in critical areas are improved. The works mentioned in the section contribute in various ways to our proposal in the field of perimeter security in critical areas; for example, the results on key exchange and the efficient implementation of cryptographic algorithms on devices with limited resources are relevant to our proposal, since it allows us to consider optimal and secure solutions to protect communications in critical areas where resources may be scarce (Lyu et al., 2021a). In addition, research on cryptographic accelerators and improving cryptographic operations in real-time is invaluable for our proposal since it guarantees a fast and efficient response in protecting communications in critical areas (Falco et al., 2018).

The works that address the detection of failures and the mitigation of possible vulnerabilities in cryptographic schemes are essential for our proposal since it helps us to identify and address potential weaknesses in the implementation of perimeter security in critical areas. Analysis of attacks and vulnerabilities, such as the copy-paste attack mentioned in one of the papers, provides essential insights to strengthen our proposition and ensure we are prepared to deal with potential threats in critical areas (Wang et al., 2019; Duan et al., 2021). Research on reliable error detection schemes and improving communication integrity is also relevant to our proposal, as it allows us to implement robust error detection and correction mechanisms in critical areas.

### 2.3. Authentication and access system through face recognition to the data center

Currently, access to the data center does not have any security. This has caused any student, teacher, or person outside the career to access the academic data center without control. This has caused security events such as equipment shutdown and manipulation of communication links, among others. The purpose of the educational data center is for students to face a natural environment and be able to manipulate the equipment under the supervision of a teacher; however, since it does not have a control system, this has become a critical point for the institution (Singh and Prasad, 2018; Villegas-Ch et al., 2022).

As a control measure, creating a system that oversees access through a face recognition algorithm is established. With this process, the aim is to automate the authentication method and assign roles and profiles based on the systems available to the institution. For the design of the authentication method, deep learning techniques are used, where the following stages are established:

- Data Collection Firstly, facial image data is collected from authorized and unauthorized persons. The more data you have, the better the model's ability to recognize faces.
- Data preprocessing, image data can be normalized, cropped, and scaled so that all images have the same size and orientation. Data augmentation techniques can also be applied to increase the amount of data and avoid overfitting.
- Model selection, a deep learning model is selected that is suitable for the facial recognition task. For this, CNNs algorithms are used, which have been shown to perform highly in image classification tasks.
- Model training, the model is trained using the preprocessed image data. The training can be carried out using a supervised learning algorithm, where the correct labels of each training image are provided.
- Model validation, a validation data set is used to measure the accuracy and effectiveness of the model. Proof can help determine if the model is overfitting or underfitting and can help tune hyperparameters to improve model performance.
- Model evaluation, once the model has been trained, its ability to recognize faces is evaluated. This can be done using a test data set not used during training or validation.
- Implementation of the model; finally, the model is implemented in a real-time authentication system. The system may use a camera to capture the user's facial image and compare it with the photos in the authorized database to determine whether access is granted.

**Figure 1:**
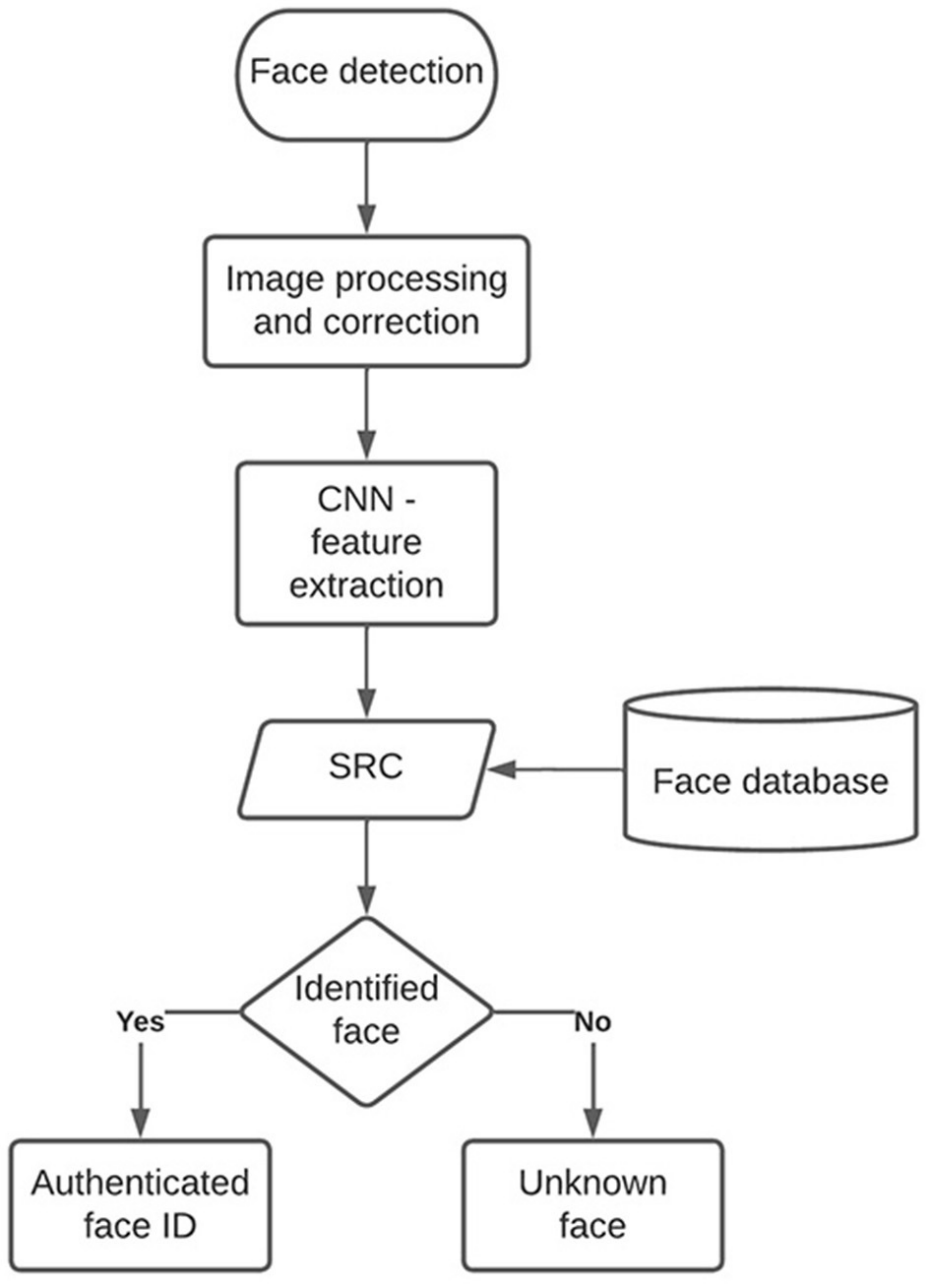
Flowchart for face recognition and identification with convolutional neural networks.

Figure 1 shows the flowchart that refers to the stages included in the algorithm's design for face identification. Two steps are passed to develop the authentication and access system; In the first, facial detection is used. This stage consists of searching for a face within an image or a frame in case a video is used. In the second stage, facial recognition is carried out; the algorithm uses AI classification techniques on the detected face to search for and identify a person (Tarlak et al., 2016; Ruz et al., 2020). To carry out this process, a database of images of the people to be determined is necessary, as well as a classifier trained for recognition.

#### 2.3.1. Image processing and correction

For face recognition, it is necessary to have an image base that contains the faces of the people who will have access to the area to be controlled. Therefore, it is essential that when collecting mugs, specific parameters are considered that help improve the efficiency of the system. One of the parameters is various facial expressions, such as happiness, sadness, stress, annoyance, etc. In addition, it is significant that the faces include as many variations as possible, such as glasses, different hairstyles, winks, and closed eyes (Ortiz-Garcés, 2019; Adusumalli et al., 2021). Another fundamental parameter is the environment where the images are obtained, which includes variations in lighting conditions, different places, the place of control, different cameras, etc. Regarding the number of ideas for the development of the system, this is a parameter that is not defined. Since no guide fits all possible environments that include computer vision, the only variable that can be considered is the more significant number of images and their variety, which improves the algorithm's performance.

To improve the acquisition of face data, the technique asks the participants for a video of their faces where parameters such as expressions and the environment are considered. From the requested video, by software, it is possible to obtain the frames and create a database with the faces of the users quickly and in quantity considered necessary; for this work, 450 images per user are established. For the development of this work, Python is used as a programming language, initially for the acquisition of faces; various AI and image processing libraries are used, such as OpenCV, imutils, face_recognition, etc.

#### 2.3.2. Feature extraction with convolutional neural networks

**Figure 2:**
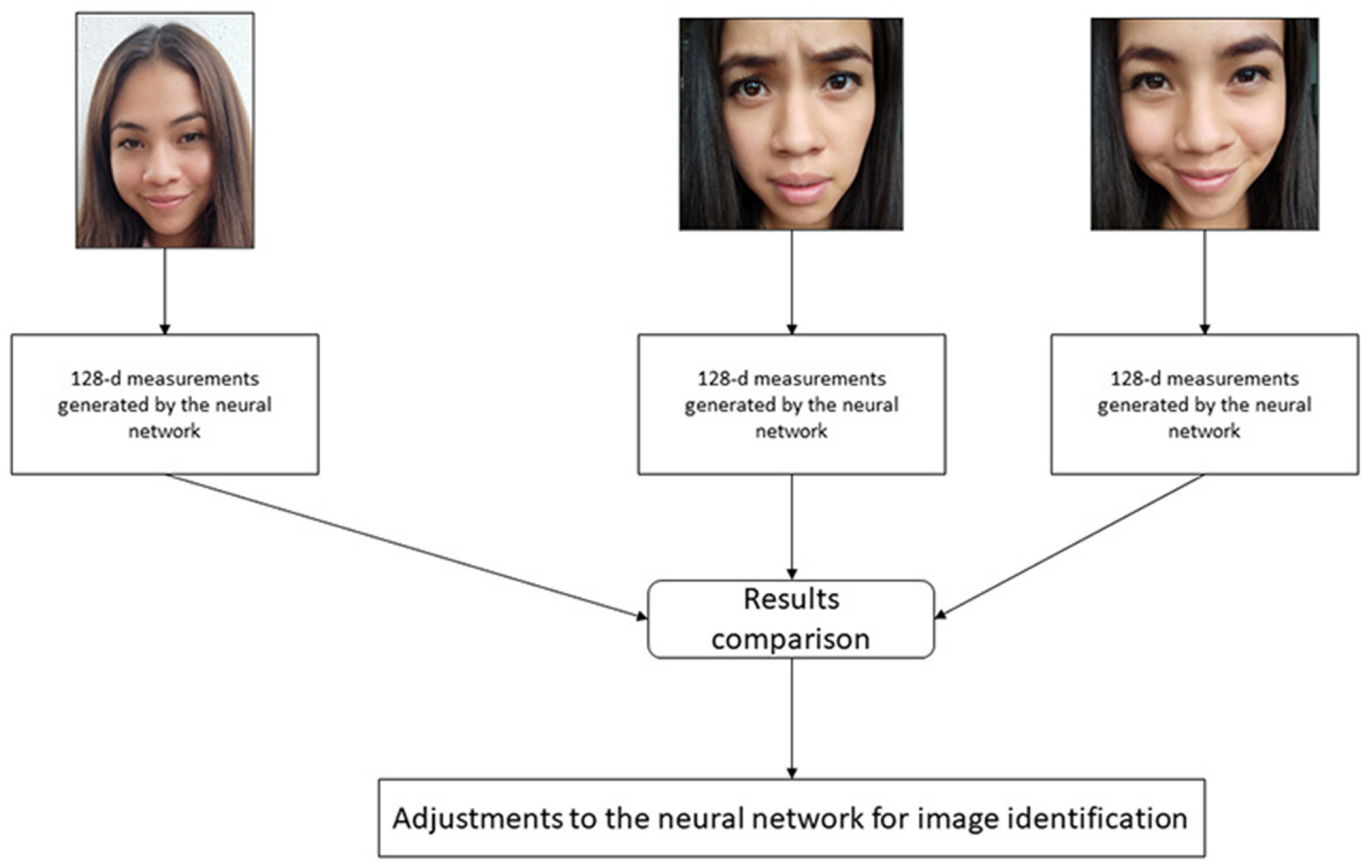
Slight adjustments to the neural network so that the measurements of the two images are closer and the measurements of the third image are further apart.

For the identification of faces, the deep learning facial recognition model is used through a technique known as metric learning. Typically, a neural network is trained to classify or generate a label for a single input image when working with deep learning techniques. However, deep metric learning works differently; instead of developing a single brand, it generates a vector of real-valued features, which guarantees the identification of a face. The dlib library is used for facial recognition and feature vector generation. This library contains efficient functions, ratios, and algorithms for working with images and faces. Using this library, a knowledge network is generated with a vector of output features of 128 numbers with actual values (128-d) responsible for quantifying a face for training; dlib uses triplets. The triplet consists of three unique facial images, as shown in Figure 2, two of the three belong to the face of the same person; the neural network generates a 128-d vector for each face image (Aghaie et al., 2016; Zhu and Cheng, 2020). For the two photos of the beginning, the neural network weights are modified to bring the vector closer through the distance metric.

The algorithm analyzes the measures generated for each of the three images. It adjusts the neural network slightly to ensure that the criteria developed for images two and three are closed, while the standard for images one and two are further apart. This process is repeated millions of times for “N” images of thousands of people. With this, the neural network learns and guarantees the generation of 128 reliable measurements for each person. This technique is known as inlays to the measurements of a face. The training of the convolutional neural network and the generation of embeddings requires a large volume of data; it can even require a high demand for resources (Azarderakhsh et al., 2016). However, once the network has been trained, it can generate measurements for any face, even if it has never processed them.

The implemented algorithm focuses on face identification using facial recognition based on convolutional neural networks. First, the dlib library is used to generate 128-dimensional feature vectors, known as embeddings, that uniquely represent the distinctive features of each face. These embeddings are obtained through a training process based on metric learning, where a neural network is trained so that the embeddings of images of the same individual are closer in feature space than those of images of different people.

Once the embeddings are generated for a database of known people, the algorithm can identify faces in real-time. When an image of a person is captured, its embedding is extracted using the same process used during training. This embedding is then compared with the embeddings in the database to find the closest match. A Simple Linear Classifier (SLC) or other basic classification algorithm is used to determine the captured person's identity. The classifier compares the similarity of the embedding of the captured face with the embeddings of the known people in the database. If a high enough match is found, it is classified as the matching person, and an institution-generated ID is provided. Otherwise, if no close match is found, access is denied.

It is essential to mention that the complete and detailed code of the algorithm is available in Appendix A of the document, where the pseudocode or actual code used to implement this algorithm is presented. This code includes the specific functions of the dlib library and possibly other related libraries.

#### 2.3.3. Face identification

This algorithm development stage focuses on identifying a person from the coding performed. The process is simplified by searching for a person in the database of known people who has the closest measurements to the image captured by a camera. A SLC is used for this part of the process. However, any basic classification algorithm can be used for this process. To find the person's name, the classifier is trained to take measurements of the input image and present the positive result if a known person's face is the closest match and present the name with an identifier generated by the person organization where you work, etc.

**Figure 3:**
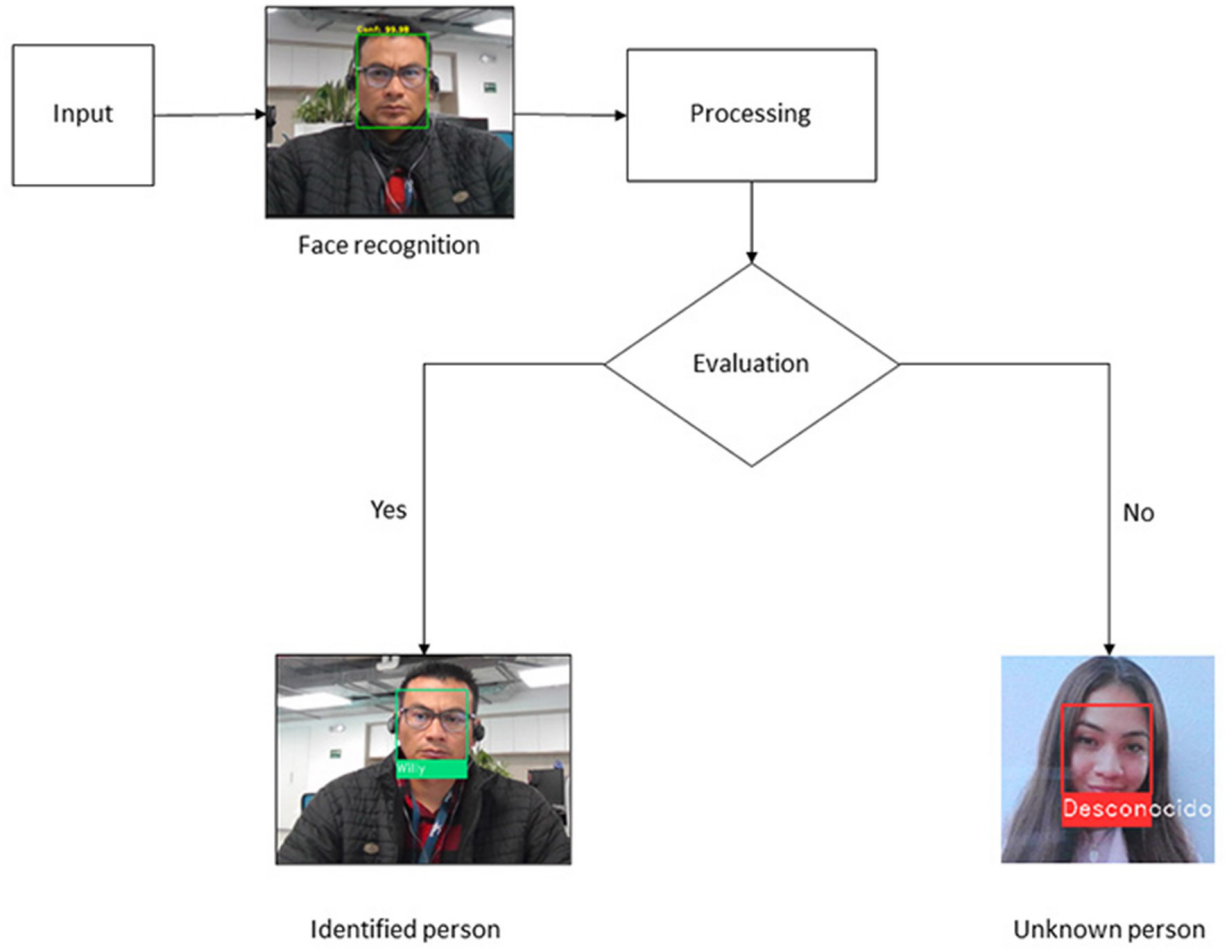
Stages for the classification of images with positive or negative identification.

For the execution of the algorithm in the authentication system, face recognition is linked to an ID generated by the institution where this work is carried out. If the ID is correct, the system authenticates the user and gives them access to the controlled area, disabling the monitoring system (Zelinsky, 2009). Log data is stored in a database that can be accessed to generate logs and reports. Figure 3 represents the face recognition process and the identification of a person with the recognition algorithm implemented; if the algorithm does not identify the person, access to the area is denied.

A problem identified in authentication through facial recognition is that most algorithms do not have a method to determine whether it is a person or a photograph. For its part, the proposed system considers this weakness and presents a solution that focuses on strengthening authentication in real-time, and that is a person. Differentiating whether it is a person, or a photograph is a challenge in image processing, but some methods can help achieve this. A deep learning model trained to distinguish between images of real people and pictures can be used to classify new images. For example, a pre-trained CNN such as InceptionV3 or VGG16 can extract features from images. A dense layer can then be trained on top of the model to classify the images as “person” or “photograph.” To prepare this model, you would need a dataset containing images of real people and photographs. Another technique that can be used is image tamper detection. Some features that can be looked for in an image to determine if it has been tampered with include inconsistencies in lighting, perspective, or depth of field. You can also look for watermarks, jagged edges, or strange patterns on the image that indicate it has been tampered with. Appendix B presents a part of the code for identifying a person or a photograph, for which the Keras library and a pre-trained model of the CNN VGG16 are used. The pre-trained model VGG16 extracts feature from the image to be classified. The predict_image function loads an image and processes it to fit the input format required by the model. The model then predicts whether the image is a person or a photograph.

### 2.4. Critical areas monitoring system

A computer vision monitoring system for critical areas is an advanced technology that automatically detects abnormal events and situations in industrial, security, and surveillance environments. It uses high-resolution cameras and image processing algorithms to analyze the content of captured images in real-time and detect moving objects, people, and vehicles. The system can be configured to generate alerts in case of suspicious activities or risk situations, allowing operators to take quick action to ensure the safety of people and the protection of assets. In addition, the system can be used to keep a historical record of captured images, allowing for later review of events and identification of patterns (Bayat-Sarmadi et al., 2013). Therefore, a computer vision critical area monitoring system is valuable for improving safety, efficiency, and asset protection in various environments.

Monitoring in data centers with computer vision allows automatic detection of events and abnormal situations; the system can be configured to detect problems in data center equipment, such as abnormal temperature, humidity, and dust, and generate alerts so that operators can take preventive measures to avoid possible failures (Zhao and Ge, 2013). Additionally, it is a valuable tool for improving security, efficiency, and asset protection in a critical business continuity environment.

Several steps are involved in implementing the computer vision data center monitoring system. It is important to note that these steps can vary depending on the system's complexity and each data center's specific needs.

- Identify system requirements; it is essential to understand the specific monitoring needs and the critical areas of the data center that must be monitored.
- Hardware selection, select the high-resolution cameras and sensors needed to capture images and detect anomalous events.
- Software development, develop image processing algorithms and analysis software to detect events and generate alerts.
- Integration, integrate the monitoring system with the data center's existing security and supervision systems.
- Testing and validation, carrying out exhaustive system tests to ensure its correct operation and adjusting if necessary.
- Staff training, train data center staff to use the monitoring system and its correct response in case of alerts.
- Maintenance, schedule periodic maintenance to ensure the system's proper functioning and update it when necessary.
- Constant monitoring, constant monitoring to detect abnormal events and take corrective measures.

#### 2.4.1. Algorithm for monitoring critical areas

**Figure 4:**
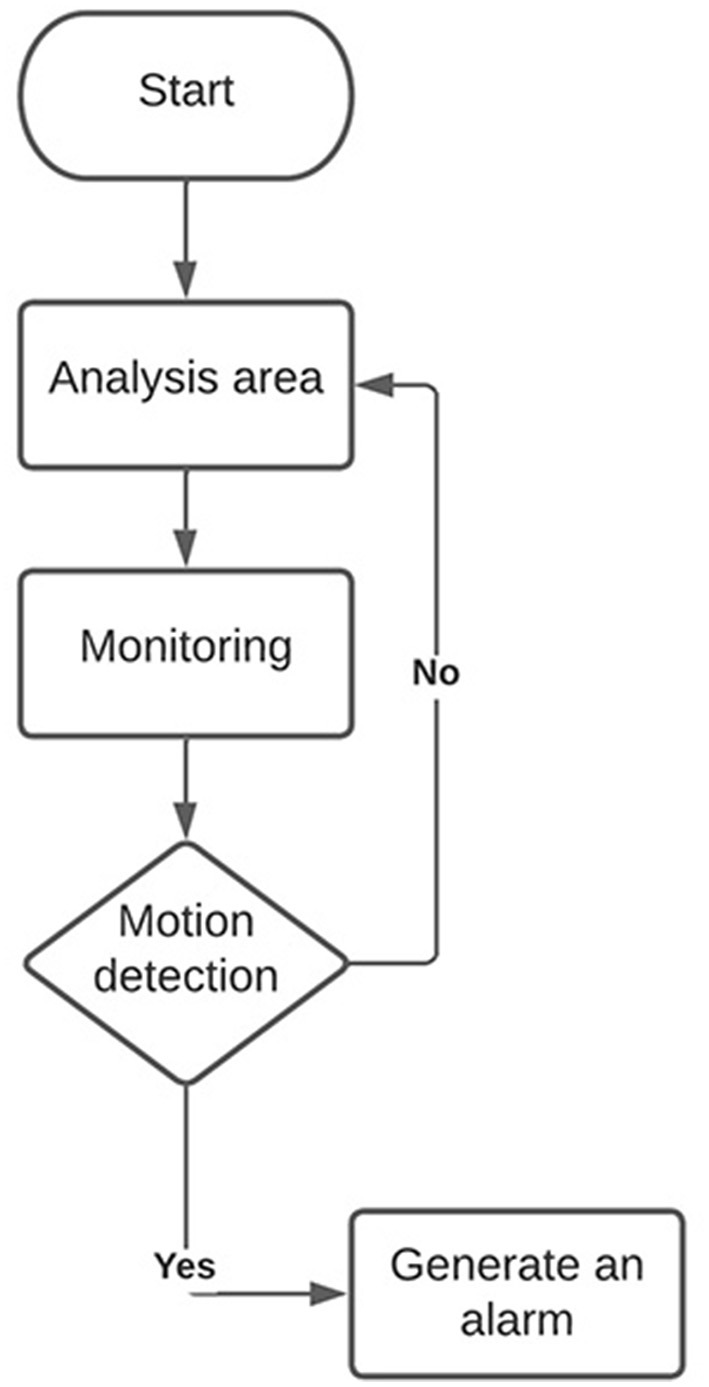
Flowchart for monitoring critical areas.

The design of the algorithm for monitoring critical areas involves several steps and techniques for its design, such as the selection of image processing techniques. This involves selecting the appropriate image processing techniques to detect the desired events. For example, object-tracking techniques, motion detection, pattern analysis, etc., can be used. It must be ensured that the algorithm can detect the desired events with a high degree of precision and a low level of false positives. Integrating the algorithm into the complete monitoring system and ensuring it works correctly with the selected hardware and software. There are different tools and libraries in Python for image processing and monitoring algorithm development, each with advantages and disadvantages. It is essential to choose the appropriate libraries for each specific case according to the needs of each organization. In Figure 4, a flowchart is presented with the monitoring system's activities. For developing the critical areas monitoring system, motion detection is used in a video sequence using the OpenCV library in Python. The algorithm uses the BackgroundSubtractorMOG2 technique to create a model of the scene's background and then applies this model to each frame to detect changes in motion. A binary mask marks the areas where activity has been detected, and a rectangle is drawn around these areas in the original frame.

**Figure 5:**
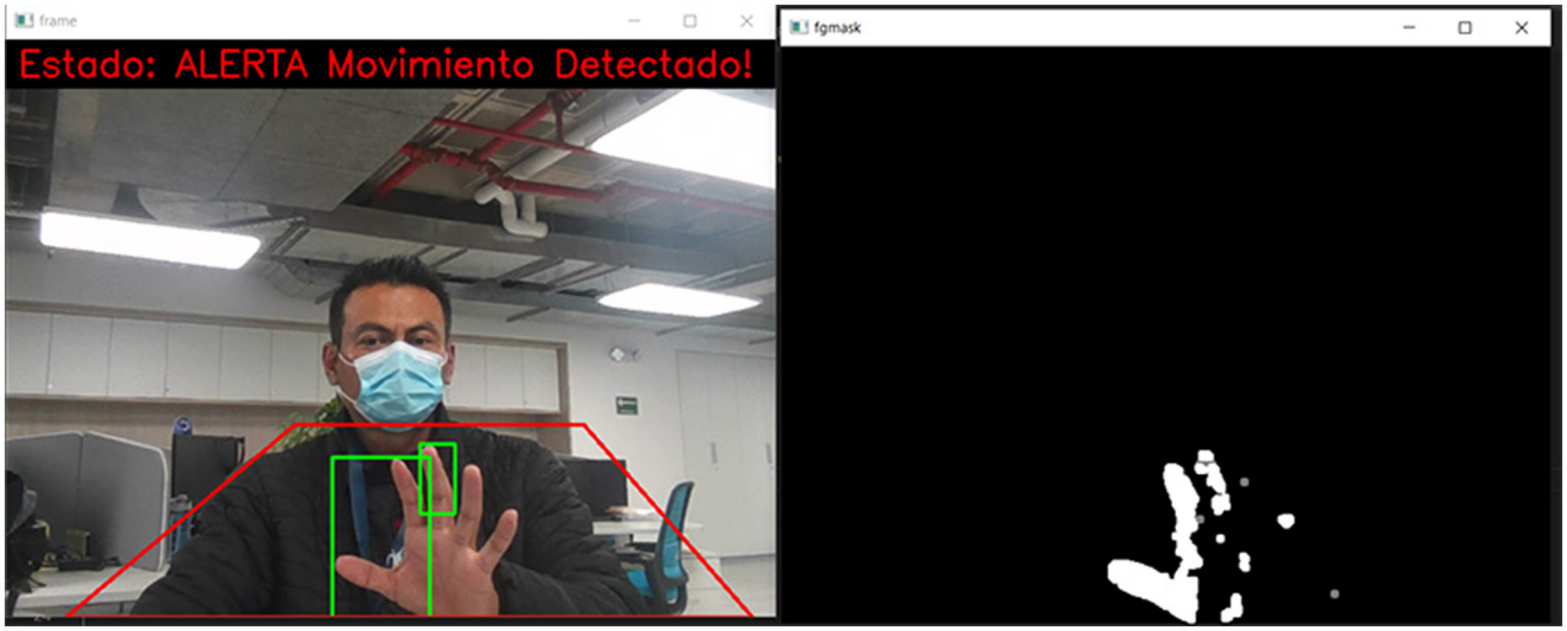
Motion detection with conversion to binary images (the notifications of the images belong to the captures made by the system, these are kept in Spanish, since this is the native language of the place where this work was developed).

The main idea of using the background subtractor is to obtain a movement mask, where the white pixels represent the presence of movement and the black ones represent the absence of action, as shown in Figure 5. For that, the apply (image_area) function is used on the gray image of a specific frame. The mask is then processed with a series of morphological operations to improve motion detection, and contours are searched on the show to determine whether there is motion. Next, the cv2.getStructuringElement() library creates a structuring kernel to apply a morphological operation to remove noise and improve motion detection. Finally, the final frame with motion detection is displayed in a window (Fernãndez et al., 2013; Sigut et al., 2020).

### 2.5. Facial recognition system application for data center access

The system is applied in a data center of an institution, considering that it requires an improved security system to control the access of authorized personnel. The objective of the application seeks to implement a facial recognition system that accurately identifies authorized users. Improve system correct identification rate to reduce false negatives and increase data center security by preventing unauthorized access.

The facial recognition system must meet specific functional requirements (RF), such as:

- RF1: Capture high-quality facial images using a high-resolution camera.
- RF2: Extract and store the unique facial characteristics of authorized users.
- RF3: Compare the facial characteristics of a person with the stored characteristics to determine their identity.
- RF4: Allow access to the data center only to authorized users.
- RF5: Record access attempts, including both successes and failures to identify.
- RF6: Provide an administration interface to add, delete or modify authorized users.
- RF7: Notify security personnel in case of unauthorized or suspicious access attempts.

Non-functional requirements include:

- RNF1: The system must have a correct identification rate >95% for authorized users.
- RNF2: The identification and decision time of the system must be < 1 second.
- RNF3: The system must recognize users in different lighting conditions, postures, and appearance changes (for example, use of glasses, beard, makeup).
- RNF4: Captured facial images and user data must be stored and managed by privacy and data protection regulations.
- RNF5: The system must be easy to maintain and update, allowing the incorporation of new technologies and improvements in the future.
- RNF6: The system must handle a growing database of authorized users without compromising performance.

The proposed system is based on facial recognition algorithms and machine learning techniques. A high-resolution camera will capture sharp facial images, which will be processed and compared with the facial features stored in the database. Error detection and correction techniques are implemented to improve accuracy, and adjustments will be made to the confidence thresholds of the recognition algorithm to minimize false negatives. The facial recognition system is implemented following the following steps:

Data acquisition and preparation:

- High-quality facial images are acquired to build a database of authorized users.
- Images are preprocessed to remove noise, normalize lighting, and extract relevant facial features.

Development of the facial recognition model:

- A facial recognition model is trained using machine learning techniques like CNN or facial representation models.
- The model is trained using the facial images of authorized users, optimizing their identification capacity, and reducing false negatives.

System implementation:

- A user interface for the facial recognition system is developed.
- Algorithms for the detection and comparison of facial features are implemented.
- The system is integrated with the high-resolution camera and existing hardware in the data center.

Tests and evaluation:

- Extensive system testing uses a diverse test data set, including different lighting conditions, poses, and appearance changes.
- The system's performance is evaluated in accuracy, response time, and compliance with the established requirements.

## 3. Results

For the results, the method was applied in a controlled environment, this being the academic data center of the university participating in this study. The data center initially had free access, with a single control by the teacher in charge of a subject where they used it. This brought with it many problems in the perimeter security of the data center. To answer these problems, an AI algorithm is created, which oversees the security control of the entire data center.

The first algorithm is responsible for authentication and access to the data center; computer vision techniques are used for this. Among the hardware used is a dome-type IP camera as the primary device; this camera is not top about its characteristics since the use of hardware that is part of the university has been arranged as a priority in this work without including older costs and giving greater weight to software performance. Therefore, among the reference characteristics of the camera is that it is IP, night vision, waterproof, and has a resolution of 4 MP.

For the detection process, the camera is installed outside the data center, and it oversees the recording of the faces of the people who want to access the cold room where the data center is located. The algorithm is always available. First, it performs face recognition in the environment; if a face exists, it compares it with its image base and identifies the person. In the next stage, the system takes the data of the identified person and contrasts it with the authentication system database. In the first stage, this system is managed by a radius; in the second stage, the authentication and access system will be integrated into the active directory of the university. Finally, in the classification, if the identified person's name is in the Radius system, the roles, and permissions that he has been verified, and the system gives him access to the data center, this is done through an automated closing and opening of system doors.

**Figure 6:**
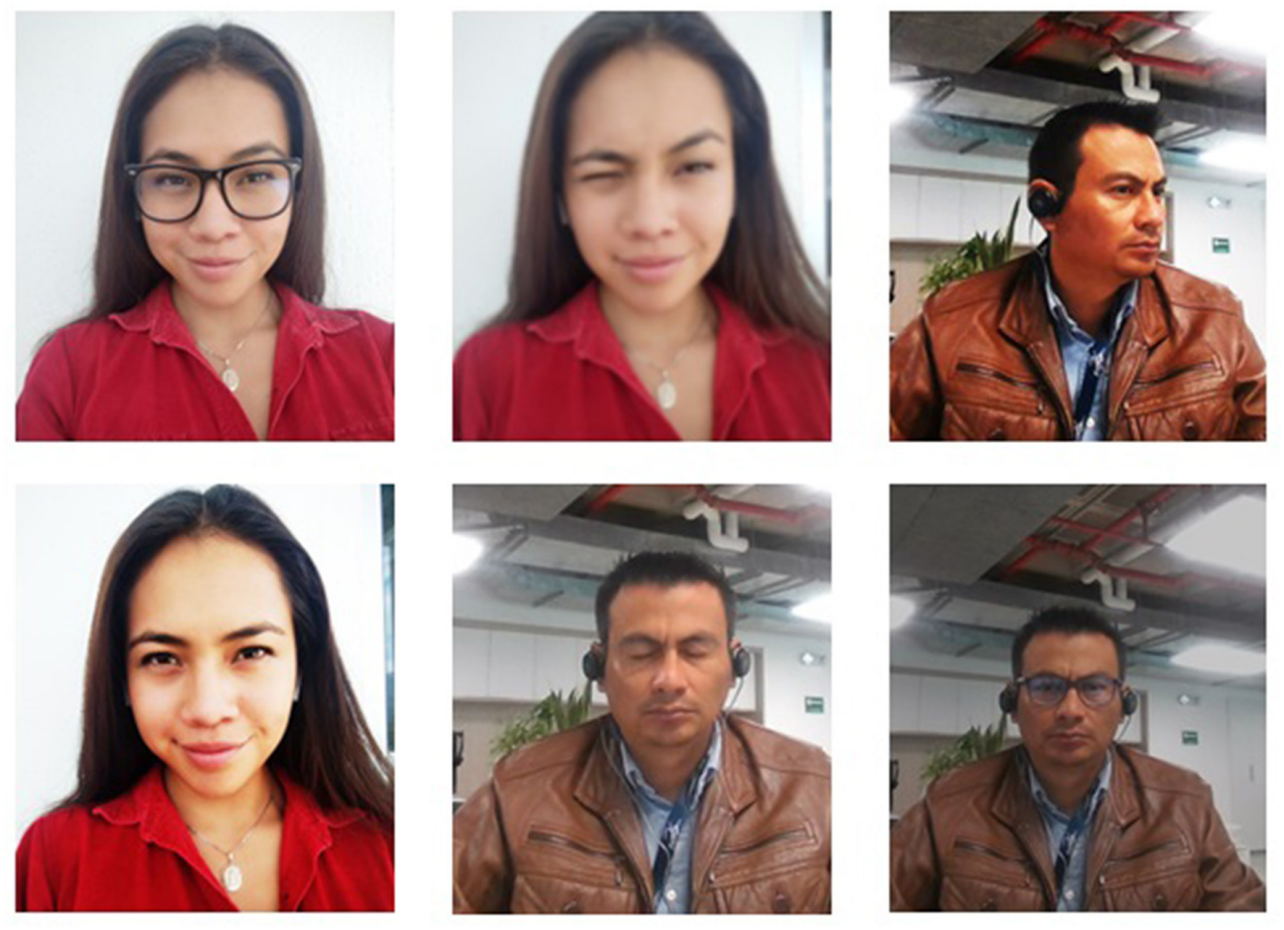
Capture of images for the population of the database used for the training of the neural network.

For this part of the system, the following tests were carried out; the registered population is 62 people, 58 students and four teachers. Of the 62 people, 27,900 images have been generated, corresponding to 450 for each user. These images have been developed using a video requested from each person and using the algorithm; the corresponding frames are obtained. Figure 6 presents a sample of the images that are part of the database used by the system; as can be seen, in the pictures, different lighting is searched for, as well as a variety of people's gestures; people were even asked to make use of glasses and changes in their hairstyle, this to guarantee the training of the classifier.

The algorithm, already in its execution, performs the following process:

- First, a particular image is read, and face_recognition is used to find its location and facial encoding.
- A video stream is then started from the webcam, and face_recognition is used to find the locations of the faces in each frame.
- For each face found, its face encodings are compared with the face encoding of the previously read image, and it is determined whether the look is known or unknown.
- Finally, rectangles are drawn around the faces, and names (user_name or Unknown) are written on the image and displayed in a window.

When using face_encoding, it generates a feature vector of 128 elements for each face. This vector is significant since it is used to compare faces. Table 1 shows the first nine results of the vector, which are the image data that will be purchased with the input video.

**Table 1:** Values of the vector generated in the comparison of the input image with the training database.

**Vector**		
−9.92080048e-02	8.86999518e-02	3.42667624e-02
3.99215659e-03	1.82419736e-02	−1.27745569e-01
−1.62490010e-02	−8.72227326e-02	1.66125849e-01

**Figure 7:**
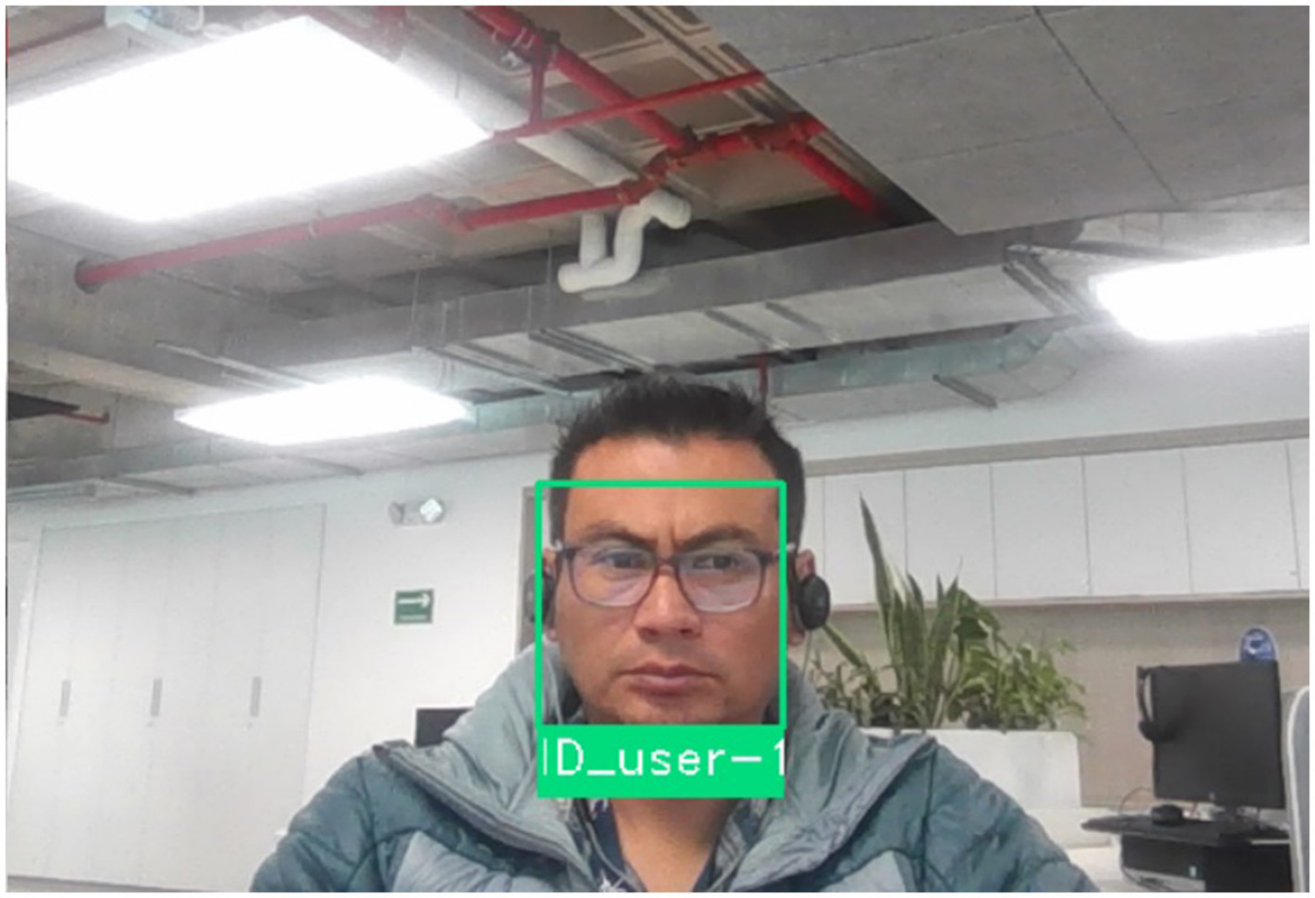
Positive identification of a user through the authentication and access system.

Once the algorithm compares the vector with the input face, the result is the name if found in the database. Otherwise, the result is unknown. In Figure 7, the development of the comparison and identification of faces is presented; in this case, the effect is positive. Therefore, the user is identified, and the system allows access to the data center. Due to the handling of personal data, ID_User-1 has been placed in the figure as a name reference.

Table 2 shows the tests carried out, these were carried out in five sessions where 62 users were considered, and in each session, five authentication attempts were recorded. The table presents the results in two groups; in the first, the adverse effects are presented, and in the second group, the positive results are recorded. The mechanics of the tests are carried out based on the confidence value of phase_recognition; the library takes the comparison of an image with the database as positive, provided that the value is >6. The confidence value calculations focus on obtaining a numerical representation of the faces (embeddings) and quantifying the similarities between them. One way to calculate this similarity is using the Euclidean distance or the cosine distance between embeddings: the smaller the space, the greater the likeness of the faces. For registration, the user approaches the access camera that has been placed 1.80 meters above the ground at the entrance of the data center. The algorithm is always online; therefore, if a person approaches a maximum distance of 60 centimeters, the algorithm can detect the face since that distance is within the range of execution of the system.

**Table 2:** Results obtained from the registered accesses of 62 users for the adjustment of parameters of the authentication system.

	**Session 1**	**Session 2**	**Session 3**	**Session 4**
Negatives	32	16	7	2
	32	14	2	1
	33	14	8	0
	27	19	6	1
	38	23	6	1
Total	162	86	29	4
Positives	30	46	55	60
	30	48	60	61
	29	48	54	62
	35	43	56	61
	24	39	56	62
Total	148	224	281	306

For the tests, a first session was considered where a webcam for domestic use and conventional lighting were included (no additional lights were modified or added); the confidence value was not changed. In this environment, 162 negative actions were obtained; of the 310 records, 52% attempted the algorithm that did not recognize the users, with 48% of access allowed. In the second session, the lighting was improved by integrating white light into the entrance to the data center. As a result, two points increased the percentage of the algorithm's confidence value. Making these changes resulted in a significant increase in positive user access results. The rate of negative accesses where the system did not recognize the users dropped to 28%, with 86 negative accesses and 224 positive ones. For the third session, a change was made in the hardware, specifically in the access camera; a 2.0 MP 1080P HD camera was chosen; this camera is bullet type and with network access via RJ45. With this change, the percentage of negative identifications settled at 9%, with 29 unrecognized users and 281 positive accesses. Even though the number of correctly identified users represents 91% of the time the system was tested, it is still not adequate since changes such as the use of glasses and gestures such as closing the eyes still affect the effectivity of the user. Algorithm. Therefore, in the fourth session, the confidence value for the classification and search of the algorithm was adjusted to 7.5. To this adjustment, the system was migrated to a virtual machine with higher features; among the most important is the use of 64 GB in memory with four cores for processing. These changes made it possible to reduce to 1% the number of cases that were not identified, with only four attempts registered as unfavorable. In addition, positive accesses are favored by providing information to the user about the use of the system, for example, maintaining an adequate distance from the camera, being within a 60-centimeter perimeter of an optimal range, not covering your face with objects such as masks, scarves, etc.

**Figure 8:**
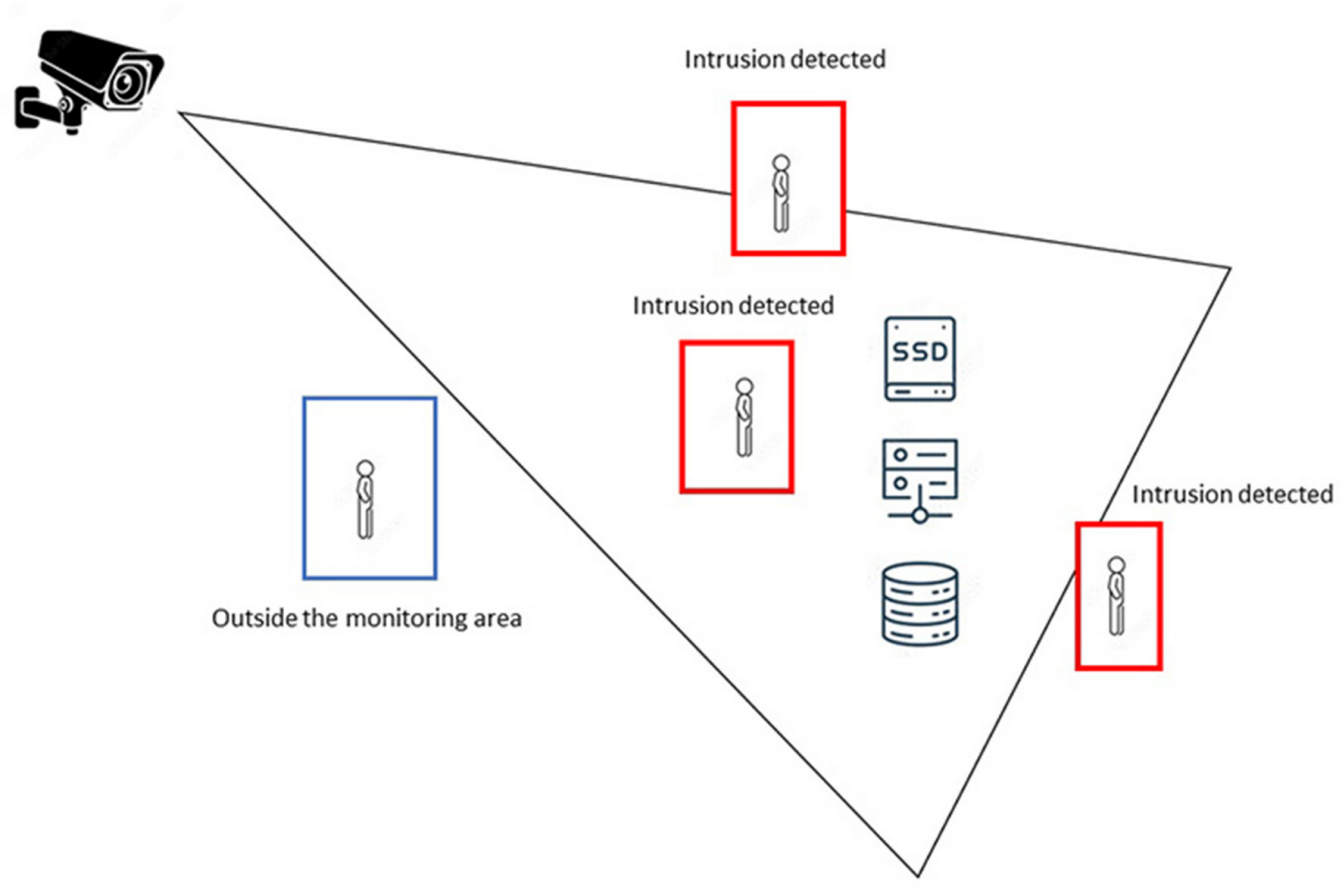
Diagram of use of the critical areas monitoring system.

The critical areas monitoring system uses the scheme of Figure 8; the components that are part of it are the camera that oversees the monitoring, the definition of the segment to be monitored, and the devices and equipment of the defined area as a critique within the institution. The figure shows the cases the system detects as violating the established perimeter. In the first instance, there is a blue box with a user outside the monitored frame that does not influence the system. Three possible cases are described below; the first is when the system slightly detects a violation of the structure; on this occasion, the subtractor slightly detects the movement; this can even occur if the air conditioning system of the data center has an extreme variation that can move a light object such as a loose cable, a sheet of paper, etc. In this case, the priority of generating alarms must be measured according to the needs of each organization. In this work, the system has been configured so that all movements detected by the subtractor create a notification. In the following two cases, where the subtractor sees a large amount of exercise, either because the person ultimately entered the monitored perimeter or there is a majority entry of the object, the system emits a notification and an audible alarm that alerts those in charge of the control center data.

**Figure 9:**
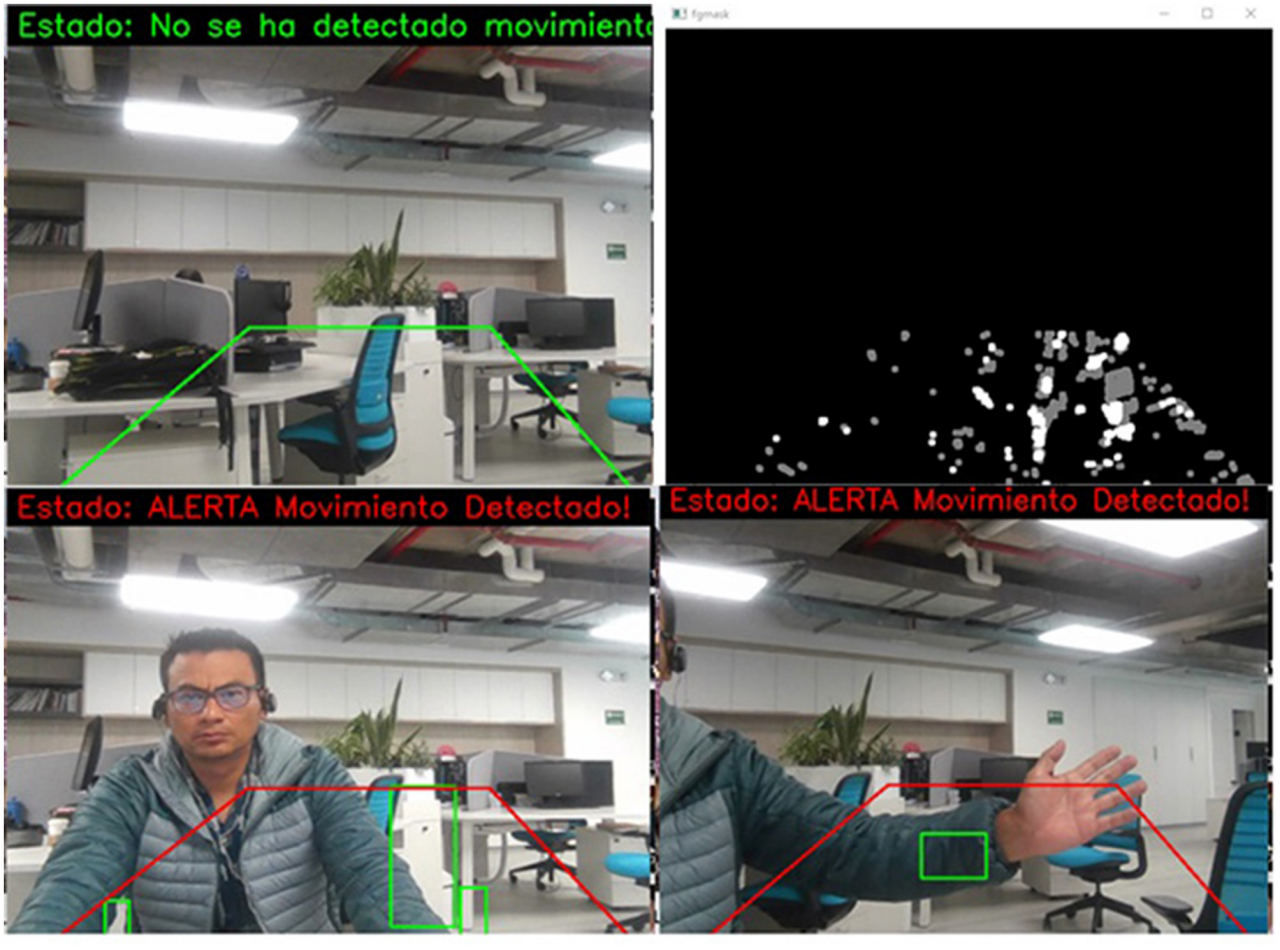
Online detection of the critical areas monitoring system, based on motion detection through a binary transformation of images (the notifications of the images belong to the captures made by the system, these are kept in Spanish, since this is the native language of the place where this work was developed).

In Figure 9, several examples of the tests carried out on the system can be seen, in which it is highlighted that the system marks a perimeter in a green box; this will change to red if there is a violation of the frame. Due to the process generated by the subtractor, the area kept in green must be black, which means there is no movement. However, when the system detects movement in the form, the blank object can be observed, which indicates movement detection, leading to the generation of an alarm. In the lower boxes of the figure, it is observed that the perimeter has been violated, and the area is drawn in red; in addition, the detection status is printed on the screen in binary. In the same way, the lower correct box shows how a movement is registered in the area. In this case, the user's arm enters the monitoring perimeter, and the system detects the motion and draws the object's outline in green.

The monitoring system went through a testing period of 15 days, during this period the results were as expected. By counting the access system as the first line of defense, this system did not find major drawbacks, however, the adjustments of the camera location are important aspects to consider. In addition, during this period there is a case in which monitoring can be negative, this occurs when a person enters the perimeter and remains still. The system, being based on movement detection, can take this action as a false negative, that is to say, that the system detects the movement in the first instance, but if the person remains completely still, the system notices the absence of an intruder even when if it is present This error can be solved with the integration of other perimeter security systems, in this work, as it is integrated with the authentication and access system, it provides the entire environment with a guaranteed level of security.

## 4. Discussion

The use of AI in perimeter security is gaining ground compared to the traditional security model. AI enables more accurate and efficient edge monitoring, helping to detect and prevent potential threats faster and more accurately (Yuan et al., 2017; Huang et al., 2020). The standard perimeter security model uses cameras, sensors, and other electronic devices to monitor a perimeter. However, these systems are often expensive, require a great deal of maintenance, and are often incapable of detecting all threats. In addition, constant monitoring requires many personnel, which can also be expensive.

AI, on the other hand, allows for more accurate and efficient monitoring of a perimeter. This is because AI uses machine learning algorithms to analyze vast amounts of data and detect patterns or abnormal behavior that may indicate a threat. This means that AI can detect threats that human personnel may not be able to see (Goncharenko and Lachihina, 2022; Xu et al., 2022). In addition, AI also enables real-time monitoring, which means that threats can be detected and prevented much faster than with the traditional model. This can be especially useful in emergencies, such as a fire or physical attack, as the AI can detect these threats and alert security personnel in seconds.

Another positive aspect of AI in perimeter security is that it is more cost-effective than the traditional model. AI requires fewer staff and less (Fraunholz et al., 2017) maintenance, which means total costs are much lower. Furthermore, AI is also more scalable, meaning it can be used in a wide variety of environments and for a wide variety of applications. However, there are also some challenges associated with the use of AI in perimeter security. For example, AI is still a developing technology, which means there may be bugs or even glitches in the system. Additionally, AI can also be vulnerable to cyberattacks, which means it is essential to have proper security measures in place (Arivudainambi et al., 2019; Steingartner et al., 2021).

This work takes computer vision systems as elements that have emerged as an alternative to traditional security models, such as surveillance cameras and motion sensors. As a result, computer vision systems offer greater accuracy and efficiency in threat detection. In addition, computer vision systems are more flexible and scalable than traditional models; they can be customized and configured to suit the specific needs of each data center (Lobanchykova et al., 2021).

The proposed computer vision system is more efficient regarding costs and resources. Unlike traditional models, which require a lot of specialized hardware and software, computer vision systems use existing hardware and software to accomplish their tasks. In addition, computer vision systems can be remotely managed and monitored, reducing maintenance costs and employee workload. However, it is essential to note that computer vision systems also present some challenges. Therefore, it is necessary to implement additional security measures to protect computer vision systems and ensure.

According to various papers (Huang et al., 2020; Juan, 2021), it is necessary to consider the possibility of image-processing attacks. These attacks focus on manipulating digital images to fool or confuse computer vision systems, including the people analyzing them. These attacks can have various objectives, such as bypassing security systems, sabotaging object recognition processes, or altering the perception of reality. Among the most common attacks are the disturbance attack, which consists of adding slight modifications to an image, invisible to the human eye, but which can change the classification of the picture by an automatic learning model. An adversary attack seeks to find an image that fools a specific image classification system (Al-Kasassbeh et al., 2020; Singh and Yow, 2021). For example, an attacker could try to generate an image that looks like a panda to a human but is classified as a gorilla by a machine-learning model. A camouflage attack seeks to hide or confuse objects in an image to avoid detection by a computer vision system. For example, an attacker could camouflage a weapon in a picture of a room to make it difficult to detect. Finally, a steganography attack is based on hiding information in an image so that it is not detectable with the naked eye (Volynets et al., 2020; Zeeshan et al., 2022). For example, an attacker could hide a message in an image that looks normal but can be discovered by a specific information extraction algorithm. It is important to note that image-processing attacks are an active field of research, and the methods used by attackers and defense systems are constantly evolving.

To mitigate them in the proposal's design, the following guidelines have been defined, generate robust training; this is a way to reduce image processing attacks by ensuring that machine learning models are robustly trained. Second, training techniques make them less susceptible to seizures (Ibitoye et al., 2019). This may include using large and diverse training data sets, model regularization, and implementing specific defense techniques. Third, attack detection can be achieved by constantly monitoring computer vision systems and implementing attack detection techniques. Data protection to prevent the possibility of manipulation by attackers. This may include techniques such as encryption of the image data and authentication and authorization of users to access it. Fourth, in people cases, people verification must involve people in the image verification process to prevent attacks. This may include using manual verification systems to verify the authenticity of images and implementing multi-stage verification processes.

In many of the reviewed works, facial recognition is approached with similar approaches, such as high-quality image capture, facial feature extraction, and comparison with a database of authorized users. However, an important distinction of our study lies in the controlled environment in which the evaluation took place, specifically in the academic data center. This allowed us to face perimeter security challenges and evaluate the system's effectiveness in a realistic scenario. In addition, our focus has been on applying artificial intelligence algorithms to monitor and improve security control in the data center, providing a comprehensive and scalable solution. Although there are similarities in the methods used in other works, our study stands out for its contextualized approach and applicability in academic or business environments where data security is a priority.

## 5. Conclusions

The use of artificial intelligence systems in the perimeter security of data centers is a topic of great importance today. With the increase in demand for data storage and processing, it is essential to protect data centers against potential threats, whether internal or external. Artificial intelligence offers a promising solution to improve the security of these centers. AI systems can perform repetitive and monotonous surveillance tasks more efficiently than humans. This reduces the workload and the risk of human error, resulting in better security. Furthermore, artificial intelligence systems can analyze vast data and detect patterns and threats humans may miss. This allows for better identification of potential hazards and a faster and more effective response.

Another benefit of using AI in data center perimeter security is customization. Systems can be configured and trained to detect and respond to specific threats according to the needs of each data center. This means that security can adapt to changes in the environment and new threats that emerge. However, using artificial intelligence in data center perimeter security also presents some challenges. One of the main challenges is vulnerability to attacks. Since artificial intelligence systems are based on software and hardware, they can be vulnerable to cyber-attacks. Therefore, it is essential to implement additional security measures to protect artificial intelligence systems and ensure data protection.

Also, artificial intelligence systems can fail. If technical failures occur, designs may not function correctly and cannot detect and respond to threats effectively. Therefore, it is essential to conduct regular tests and verifications to ensure the proper functioning of artificial intelligence systems.

Even though the results of the system are viable for its expansion, it is essential to establish that there are some limitations in the authentication and monitoring system with AI for perimeter security, among which can be highlighted. First, accuracy considering is essential for the effectiveness of perimeter security. However, the accuracy of AI systems can be compromised by factors such as the quality of surveillance camera images, lighting, weather conditions, and the presence of obstructing objects. Second, a perimeter security system must be prepared to adapt to changing problems and unknown threats in adapting to new situations. The ability of AI systems to adapt to new conditions is highly dependent on the quantity and quality of the training data used to train the model. Third, the cost is a limiting factor in implementing an AI perimeter security system in terms of hardware and software. Fourth, privacy can raise concerns, as these systems can capture and store images of people in public areas. Therefore, it is essential to establish clear privacy policies and comply with the corresponding regulations. Finally, maintenance and updating, if inadequate, can compromise the system's security and make it vulnerable to new threats.

Considering the results obtained in this study, there are several opportunities for future research in facial recognition and perimeter security in critical environments. A promising direction would be the refinement of the facial recognition algorithm to improve the identification rate and reduce the cases of false positives and false negatives. This could be achieved by exploring advanced machine-learning techniques and more sophisticated computer vision algorithms. Additionally, testing and analysis in more challenging environments, such as variable lighting conditions, changes in user appearance, and partial obstructions, would be beneficial. Likewise, strategies could be explored to integrate the facial recognition system with other perimeter security systems, such as intrusion detection or physical access control systems. These efforts would further strengthen safety and security in critical areas and improve the user experience and overall system efficiency.

## Data availability statement

The raw data supporting the conclusions of this article will be made available by the authors, without undue reservation.

## Ethics statement

Ethical review and approval was not required for the study on human participants in accordance with the local legislation and institutional requirements. The patients/participants provided their written informed consent to participate in this study. Written informed consent was obtained from the individual(s) for the publication of any potentially identifiable images or data included in this article.

## Author contributions

Conceptualization and validation: WV-C and JG-O. Methodology, formal analysis, resources, writing—review and editing, visualization, and supervision: WV-C. Software, investigation, data curation, and writing—original draft preparation: JG-O. All authors have read and agreed to the published version of the manuscript.

## Appendix

### Appendix A

import cv2

import face_recognition

# Imagen a comparar

image = cv2.imread(“C:/Users/Desktop/DetectorFace/ imagenes_ videos/image_0006.jpg”)

face_loc = face_recognition.face_locations(image)[0]

#print(“face_loc:”, face_loc)

face_image_encodings=face_recognition.face_encodings (image, known_face_locations=[face_loc])[0]

print(“face_image_encodings:”, face_image_encodings)

# Video Streaming

#cap = cv2.VideoCapture(0)

cap = cv2.VideoCapture(0, cv2.CAP_DSHOW)

while True:

ret, frame = cap.read()

if ret = = False: break

frame = cv2.flip(frame, 1)

face_locations = face_recognition.face_locations(frame)

#face_locations = face_recognition.face_locations(frame, model=“cnn”)

if face_locations != []:

for face_location in face_locations:

face_frame_encodings=face_recognition.face_encodings (frame, known_face_locations=[face_location])[0]

result = face_recognition.compare_faces([face_image _encodings], face_frame_encodings)

# print(“Result:”, result)

if result[0] = = True:

text = “William”

color = (125, 220, 0)

else:

text = “Desconocido”

color = (50, 50, 255)

cv2.rectangle(frame, (face_location[3], face_location[2]), (face_location[1], face_location[2] + 30), color, -1)

cv2.rectangle(frame, (face_location[3], face_location[0]), (face_location[1], face_location[2]), color, 2)

cv2.putText(frame, text, (face_location[3], face_location[2] + 20), 2, 0.7, (255, 255, 255), 1)

cv2.imshow(“Frame”, frame)

k = cv2.waitKey(1)

if k = = 27 & 0xFF:

break

cap.release()

cv2.destroyAllWindows().

### Appendix B

Code to identify if it is a person or a photograph.

import numpy as np

import keras

from keras.preprocessing import image

from keras.applications.vgg16 import VGG16, preprocess_ input

# Load pre-trained VGG16 model

model = VGG16(weights=‘imagenet')

# Function to predict if the image is a person or a photograph

def predict_image(img_path):

img = image.load_img(img_path, target_size=(224, 224))

x = image.img_to_array(img)

x = np.expand_dims(x, axis=0)

x = preprocess_input(x)

preds = model.predict(x)

if preds[0][0] > preds[0][1]:

return ‘Is a person'

else:

return ‘It's a photograph'

# Usage ‘example

result = predict_image(‘example_fig1.jpg')

print(result).
